# UV-responsive nano-sponge for oil absorption and desorption

**DOI:** 10.1038/srep12908

**Published:** 2015-08-11

**Authors:** Do Hyun Kim, Min Chan Jung, So-Hye Cho, Sang Hoon Kim, Ho-Young Kim, Heon Ju Lee, Kyu Hwan Oh, Myoung-Woon Moon

**Affiliations:** 1 Department of Materials Science and Engineering, Seoul National University, Seoul 151-742, Republic of Korea; 2 Institute of Multidisciplinary Convergence of Matter, Korea Institute of Science and Technology, Seoul 136-791, Republic of Korea; 3 Department of Mechanical and Aerospace Engineering, Seoul National University, Seoul 151-744, Republic of Korea

## Abstract

Controlled surface wettability for oil has been intensively studied to remove industrial oil waste or oil spill pollution from seas or rivers. In particular, external stimuli-induced special wetting materials, such as photo-responsive TiO_2_, have attracted considerable attention for oil-water separation. In this study, a novel method is reported to fabricate a nano-sponge which is composed of hydrophobic hydrocarbon and hydrophilic TiO_2_ nanoparticles for oil absorption or desorption that are responsive to UV irradiation. The hydrocarbon in the nano-sponge could selectively absorb oil from water, whereas the absorbed oil is released into the water by TiO_2_ in response to UV irradiation. The nano-sponge functionalized porous polydimethylsiloxane released more than 98% of the absorbed crude oil with UV irradiation and air-bubbling. It could be continuously reused while maintaining a high absorption capacity and desorption efficiency without incurring secondary air or water pollution. This smart oil absorption/desorption methodology with excellent selectivity and recyclability with almost perfect removal of absorbed oil can be applied for oil-water separation, oil spill cleanup and reuse of spilled oil.

Collecting oil in the ocean or other bodies of water has been considered as a challenging environmental issue. Therefore, oil collection has become a highly important research topic due to the increasing amounts of industrial oily wastewater and frequent oil spills. Several methods for oil cleanup have been developed, such as the use of oil absorbents or oil skimmers^1^,^2^,^3^,^4^. However, recovered oil by these methods typically contain at least 5 ~ 10% of water^3^,^4^. To separate oil from water, recent approaches have focused on controlling the wettability of the oil or water. Two types of structures or materials have been explored: oil removal and water removal. The oil-removing materials are required to have superhydrophobicity while possessing superoleophilicity in air, to absorb only the oil from the water under the surface by using various porous sponge-like materials^5^,^6^,^7^,^8^,^9^,^10^,^11^. However, because oil-removing materials are easily fouled by high oil adhesion due to their oleophilic nature, the reusability of the materials is limited by degraded separation or absorption capacity^7^,^8^. In the case of water-removing materials, the wettability needs to be superhydrophilic; however, the material or structure needs to be superoleophobic underwater^12^,^13^,^14^,^15^,^16^,^17^,^18^. Due to high water adhesion to the material surface, water has a very low wetting angle. The underwater superoleophobic interface with low affinity for oil drops prevents the coated materials from oil fouling, which makes the oil and the material to be easily recycled. Therefore, this water-removing property has been applied to oil-water separation filters^13^,^14^,^15^,^16^,^17^,^18^.

Recently, it has been reported that surface wettability can be switched or tuned using external stimuli such as light irradiation^19^,^20^, mechanical loading^21^, chemical treatment^22^, electric fields^23^, magnetic fields^24^, temperature^25^, and pH^26^,^27^. In particular, switchable wettability between superhydrophilicity and superhydrophobicity has been studied on various photo-responsive materials such as TiO_2_^28^,^29^,^30^,^31^,^32^, ZnO^19^, and SnO_2_^20^. The switchable wettability of TiO_2_ has been reported. Its original water CA of 35° undergoes a dramatic wettability transition due to UV irradiation, carendering it superhydrophilic with a CA_water/air_ less than 5°; however, the underwater oleophobicity increases, as evidenced by the change in the oil (heptane) CA underwater from 146° to 167°^30^. The role of TiO_2_ under UV irradiation is known to enhance the surface superhydrophilicity and underwater superoleophobicity. Thus, UV irradiation on TiO_2_ enables versatile applications for antifogging and self-cleaning^28^, photo-controllable oil/water filtration^30^, and water purification^31^. Although many researchers have focused on oil/water separation using photo-responsive TiO_2_, the uses of wettability conversion between hydrophilicity and hydrophobicity are limited to mesh type filtration^30^. To work as oil absorbing materials in the presence of water, TiO_2_ should have both hydrophobicity and oleophilicity. However, as-prepared TiO_2_ surface typically shows hydrophilicity, and subsequent surface modifications are needed to be hydrophobic, which degraded by UV irradiation^32^. Furthermore, a TiO_2_-coated nanocellulose aerogel was reported to show photo-induced switchable wettability^33^, illustrating that water could be absorbed and repelled with and without UV irradiation. However, the main focus of the paper is to explore the photo-induced switchable water absorption and wettability.

Here, a novel method is reported to fabricate nano-sponge materials by mixing hydrophobic porous hydrocarbon nanoparticles (NPs) and hydrophilic TiO_2_ NPs (Supplementary Fig. S1). The nano-sponge can selectively and effectively absorb oil from water due to their oleophilicity of hydrocarbon NPs. On the other hand, hydrophilic and underwater oleophobic nature due to the TiO_2_ NPs in the nano-sponge extracts the absorbed oil into water in response to UV irradiation. By mixing the hydrocarbon NPs and UV-responsive hydrophilic TiO_2_ NPs, one can control the wettability of the nano-sponge for oil and water. The nano-sponge/porous polydimethylsiloxane (NS/p-PDMS) could be fabricated by decorating the nano-sponge within the porous structure of PDMS, as shown in Fig. 1a. With the mixing ratio of the nano-sponge (the ratio of hydrocarbon NPs/TiO_2_ NPs) as 6/4, the NS/p-PDMS showed a slightly yellow color and was hydrophobic and oleophilic in air. The contact angles (CAs) for water (dyed in red) and oil (crude oil, black) were measured as 140 ± 6° and ~0°(or fully absorbed), respectively. The surface structure and the NPs distribution of the 6/4 NS/p-PDMS was analyzed in Fig. 1b,c, which show that single crystalline TiO_2_ NPs tends to agglomerate as small clusters (<200 nm) that are dispersed within and on the amorphous hydrocarbon NP networks. The distribution of hydrocarbon and TiO_2_ NPs with an average diameters of 30 ± 9 and 31 ± 9 nm (Supplementary Fig. S2), presented as carbon (red), oxygen (blue), and titanium (green) in the EELS elemental map (Fig. 1c), correspond to the bright field TEM image (inset in Fig. 1c). Through compositional analysis using energy-dispersive X-ray spectroscopy (EDS), as shown in Fig. S1, the simple mixing of hydrocarbon/TiO_2_ NPs according to their volume ratio showed a linear variation of C, O, and Ti in atomic percent (at.%). Notably, along the larger pores of the PDMS sponge that were several hundred micrometers large (inset in Fig. 1b), NS/p-PDMS showed multi-scale and hierarchical porous structures, which rendered the NS/p-PDMS to be more hydrophobic in air. Representative images of crude oil absorption/desorption through the NS/p-PDMS are shown in Fig. 1d–f. This NS/p-PDMS could selectively absorb crude oil both on the surface and under water because the sponge surface was oleophilic. As oil contacted the NS/p-PDMS, the sponge instantaneously absorbed the oil and released the air bubbles trapped inside the surface (Fig. 1d, Supplementary Movie 1). As UV irradiated, the oleophilic surface became oleophobic underwater. By introducing air bubbles (discussed later) to pass through the sponge containing crude oil (Fig. 1e), the crude oil was desorbed from the surface (Fig. 1f, Supplementary Movie 2). Overall, the process of oil absorption/desorption can be recyclable after drying in the dark, while maintaining a high absorption capacity and desorption efficiency, as described by a series of schematic illustrations in Fig. 1g.

## Results

### Tunable surface wettability under water

Because surface wettability is determined by the surface chemical composition and microstructure (Supplementary Fig. S1), changing the mixing ratio of hydrocarbon and TiO_2_ in each nano-sponge would provide different wettabilities for water or oil in different environments, such as in air or underwater. As shown in Fig. 2, the surface wettabilities of water and oil (n-hexane) on a glass slide coated with the nano-sponge were explored in air and water. The CAs for water and oil in the air were measured with respect to the mixture ratio before and after UV irradiation, as shown in Fig. 2a. By increasing the TiO_2_ content in the nano-sponge coated flat glass slide, the water CA in the air reduced from 128° for the mixing ratio of 10/0 (hydrocarbon/TiO_2_) to 0° for that of 0/10. This indicates that the surface wettability could be controlled from mild hydrophobic to superhydrophilic without UV irradiation. However, all of the water CAs was significantly reduced upon UV irradiation. In the case of ratios from 4/6 to 0/10, the water CA was almost zero in the air. In the case of oil droplets, regardless of the TiO_2_ content and UV irradiation, superoleophilicity was exhibited on all of the samples in which oils were totally absorbed by the nano-sponge in the air. However, the underwater wettability for oil dramatically changed with respect to the sample composition and UV irradiation, as shown in Fig. 2b. As the TiO_2_ content in the nano-sponge increased, the underwater oleophobicity was enhanced from 45° for the 10/0 ratio to 125° for the 6/4 ratio. The samples with mixing ratios of 2/8 and 0/10 showed superoleophobicity underwater with CAs greater than 150°, even before UV irradiation. After UV irradiation, the underwater oil CAs were also improved and all of the samples except for the 10/0 ratio (131°) showed superoleophobicity with oil CAs over 150° (see Supplementary Fig. S3 for detailed images).

The significant changes in underwater oil CA by UV irradiation were due to the changes in the surface energy of the nano-sponge. Thus, the work of adhesion or surface energy can be calculated using the Young-Dupré equation^34^,^35^ as follows:


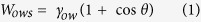


where *W*_*ows*_*, γ*_*ow*_, and *θ* are the work of adhesion in the oil-water-solid system, the interfacial tension of oil and water, and the apparent underwater oil CA, respectively. Theoretically, equilibrium CA should be used to calculate the work of adhesion. However, only the advancing CA can be reliably measured and derives minimum work of adhesion^36^. In this study, we assumed that the apparent CA is equivalent to advancing CA due to low CA differences less than 8° (Supplementary Information, Table S2). Therefore, the work of adhesion was calculated by using apparent CA, and this would show a similar trend with the minimum work of adhesion calculated by using advancing CA. Because *W*_*ows*_ is the work necessary to separate a unit area of the oil-solid interface in water, it was calculated using the interfacial tension of the oil-water and underwater oil CAs. The interfacial tension between n-hexane and water was measured as 50.2 mN/m (Supplementary Fig. S4 and Table S4), and each value of *W*_*ows*_ was calculated from equation (1) and plotted in Fig. 2b. Before UV irradiation, oil droplets adhered onto the surfaces with ratios from 10/0 to 4/6. However, oil droplets adhered only to the samples with the ratios of 10/0 and 8/2 after UV irradiation. For the other mixing ratios, oil droplets easily rolled off the nano-sponge surface under water, with a very low sliding angle (insets in Fig. 2b). As the underwater oil CA increased, the work of adhesion decreased, indicating that the interfacial adhesion of each nano-sponge drastically decreased with UV irradiation. The CAs under salt water (3.5% dissolved NaCl) were also compared with those of pure DI water, and it was found that the effect of salt was negligible (Supplementary Information, Table S3). And there was a report that 10% of UV irradiation of 305 nm can reach maximum depth of 16 m in sea water^37^. Therefore, UV induced high contrast in work of adhesion is still effective for the application in the ocean.

Because the hydrophilicity increased as the oleophilicity decreased with UV irradiation, one can suppose that water may have a greater chance to replace oil at the nano-sponge and oil interface due to the increased water affinity. Therefore, the depletion of adhesion at the interface could be explained by photocatalytic oxidation (PCO) and the wetting transition of TiO_2_ during UV irradiation^38^,^39^,^40^. X-ray photoelectron spectroscopy (XPS) measurements were performed to reveal the chemical state of the 6/4 nano-sponge before and after UV irradiation (Fig. 2c). The XPS data were analyzed for the C, O, and Ti core levels of a 6/4 nano-sponge sample. The change in the C 1s spectrum clearly showed that the hydrocarbon (C-H, 284.6 eV) was drastically decomposed into carboxyl carbon (O = C-O, 288.3 eV) and oxidant carbon (C-O, 285.8 eV). In addition, the change in the O 1s spectra corresponded to the adsorption of the hydroxyl group (-OH, 532.5 eV), which converted the surface to a superhydrophilic surface after UV irradiation^41^. Before UV irradiation, the Ti 2p XPS spectrum showed two typical values of TiO_2_ at 458.6 and 464.3 eV, which were assigned to the binding energies of Ti 2p_3/2_ and Ti 2p_1/2_, respectively. Additionally, two new peaks at 460.1 and 465.4 eV (related with Ti 2p_3/2_ and Ti 2p_1/2_) with strong intensities were found and could be attributed to the presence of strong interactions at the interfaces between TiO_2_ and hydrocarbon as Ti-O-C bond^42^. The formation of the Ti-O-C bond was also shown at 531.4 eV in the O 1s spectrum before UV irradiation, and this bond was decomposed upon UV irradiation by the PCO of TiO_2_. As a result, one can consider that the oil-hydrocarbon interface may deteriorate due to the PCO of TiO_2_ NPs. Therefore, the interfacial bond becomes very weak, and the water molecule may replace the decomposed sites of hydrocarbon NPs on the nano-sponge.

### Oil absorption and desorption using the nano-sponge

To investigate the self or spontaneous release of oil droplet due to TiO_2_ on the wetting transition and hydrocarbon decomposition at the interface, heavy oil droplets (3 M Fluorinert FC-770, density of 1.793 g/ml) were explored in contact with the nano-sponge coated glass slides with different mixing ratios of hydrocarbon/TiO_2_ (6/4 and 10/0) under UV irradiation. Heavy oil was used to show that the buoyancy of the bubble could be large enough to overcome the sum of the interfacial adhesion and the weight of the oil. The spontaneous growth of bubble within a light oil droplet, n-hexane, were monitored during UV irradiation as shown in Supplementary Fig. S5. In Fig. 3a,b, both of the samples showed mild oleophobicity underwater, with oil CAs of 125° and 113°, respectively. Due to the content of hydrophilic TiO_2_ in the 6/4 nano-sponge, oleophobicity showed slightly higher in underwater oil CA. Initially, both of the samples contained tiny air bubbles on their surfaces under water, which were captured by hydrophobic hydrocarbon NPs. Air bubbles were encapsulated by the oil immediately after the contact with heavy oil droplets, due to the positive spreading coefficient, S_o_ (+3.8 mN/m for FC-770, Supplementary Information). As UV light was irradiated, the bubbles inside of the oil droplets spontaneously grew (Supplementary Fig. S6) and dragged the oil droplet upward due to the increased bubble buoyancy. The change in the oil contact radius (*R*_*C*_) and underwater oil CA were monitored during the bubble growth under UV irradiation (Fig. 3c,d). Because changes in *R*_*C*_ were attributed to receding contact angles^43^ and interfacial adhesion, which varies with UV irradiation time on the nano-sponge, the oil and bubble desorption behaviors with varying underwater oil CA and *R*_*C*_ can be explained. In the case of the 6/4 nano-sponge, the underwater oil CA slightly increased due to the lateral bubble growth at the beginning of UV irradiation. As the bubble buoyancy increased, the lateral force exerted on the center of the oil droplet gradually increased. The underwater oil CA gradually decreased from 136° to 115°, and discrete contact line movements between the oil and the 6/4 nano-sponge surface were observed, as shown Fig. 3a. When the underwater oil CA was lowered to the receding contact angle (RCA) value, the oil droplet receded with the reduction of *R*_*C*_. The underwater oil CA gradually decreased to 85° and abruptly reduced to 59°, before the moment of oil detachment from the surface, leaving only a small amount of oil residue on the surface after 33 hours of UV irradiation (Supplementary Movie 3). As *R*_*C*_ was reduced to 0.65*R*_*C*_, necking of the oil droplet occurred close to the oil-solid interface (Inset in Fig. 3d). The neck of the oil became unstable and the oil/bubble was pinched off. In contrast, the underwater oil CA for the 10/0 nano-sponge gradually decreased from 113° to 94°, whereas *R*_*C*_was almost unchanged, as shown in Fig. 3c,d. The RCA for the 10/0 nano-sponge was expected to be smaller than that of the 6/4 nano-sponge, due to different TiO_2_ content. The decrease in the underwater oil CA in the 10/0 nano-sponge was not enough to decrease the RCA. Thus, the necking and pinching off occurred at the oil-bubble interface farthest from the oil-solid interface, leaving most of the oil droplet adhered to the surface. This phenomenon was attributed to the oleophilic hydrocarbon NPs, which firmly held the oil droplet at the interface with relatively high interfacial adhesion strength over the buoyancy of the oil-encapsulated bubble.

Regarding the forces acting at the oil-solid interface, the vertical forces of buoyancy, pressure, and surface tension are expressed in Supplementary Fig. S7. It was calculated that the remaining oil volumes were determined by the magnitude of the surface tension force acting at the oil-solid interface, before the oil or bubble detachment. Because the *R*_*C*_ for both cases were almost the same as the radius of the neck, during all of the bubble growth processes, a modification of Tate’s law can be written as follows^44^,^45^


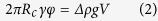


where where ρ, g, and ϕ are the density, the acceleration of gravity, and the ratio of the detached mass to the total mass, as a function of 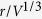, respectively. In general, 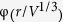 increased with a decreasing 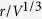 < 0.85^45^. In this case, *r* is the contact radius *R*_*C*_, and *V* is the oil volume which is constant. As a result, one can consider that the remaining oil volume on the nano-sponge surface depends on the *R*_*C*_. Reduction of the *R*_*C*_ at the detachment corresponded to the reduction of the surface tension force, which resulted in the small amount of the oil residue left on the 6/4 nano-sponge. However, a constant *R*_*C*_ for the 10/0 nano-sponge was responsible for the constant surface tension force by contact line pinning, which held most of the oil on the 10/0 nano-sponge even after bubble detachment. Overall, this oil detachment was driven not only by the buoyancy force from growing bubbles in oil but also by the reduction of the surface tension force induced by the wetting transition to superoleophobicity, further reducing the *R*_*C*_.

**Figure 1:**
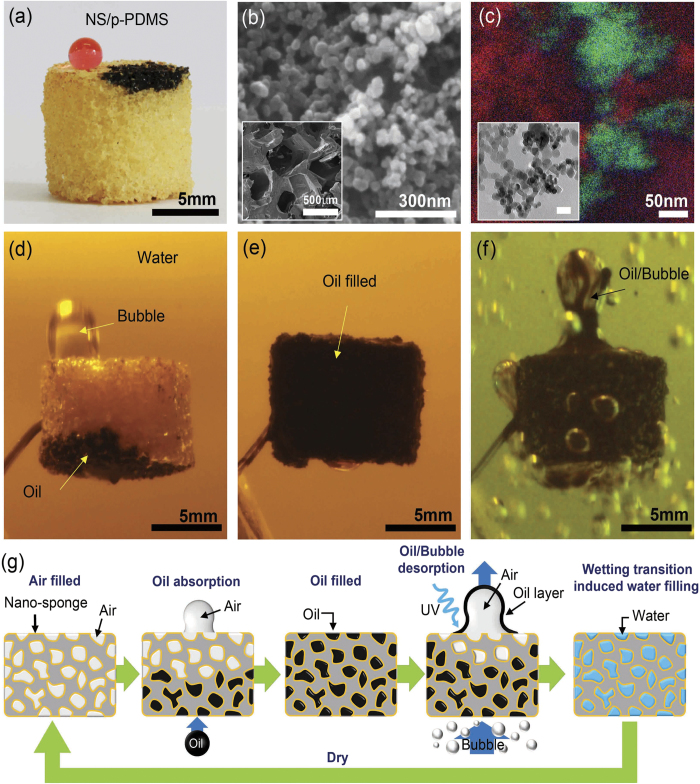
The nano-sponge (hydrocarbon/TiO_2_ NPs) and its oil absorption and desorption capacity underwater. (**a**) Fabricated nano-sponge/porous PDMS (NS/p-PDMS) by dip coating. (**b**) SEM image of the surface structure of the 6/4 NS/p-PDMS. The bright contrast and dark contrast materials are TiO_2_ and hydrocarbon, respectively. The inset shows a low magnification image of the NS/p-PDMS, indicating its porous structure. (**c**) Compositional distribution by TEM-EELS mapping; C (red), O (blue), Ti (green). The inset shows a bright field image of the 6/4 nano-sponge. (Scale bar = 50 nm) (**d**) Underwater crude oil absorption of the 6/4 NS/p-PDMS, (**e**) fully absorbed state, and (**f**) oil desorption with air bubble flow/UV irradiation. (**g**) A schematic procedure of oil absorption and desorption with UV-responsive wettability and air bubble flow.

**Figure 2:**
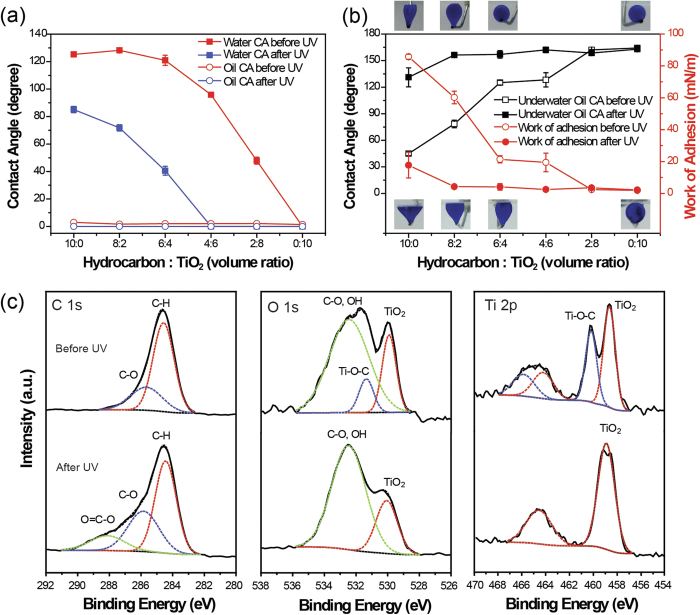
Surface wettability of water and oil (n-hexane) on a nano-sponge (hydrocarbon/TiO_2_) before and after UV irradiation. (**a**) Water and oil contact angle (CA) in air, (**b**) underwater oil CA and work of adhesion. Insets are snapshots of oil droplets immediately prior to detachment from the injection needle before (lower inset) and after (upper inset) UV irradiation. (**c**) X-ray photoelectron spectroscopy analysis before (upper graph) and after (lower graph) UV irradiation.

To visualize oil absorption/desorption with the nano-sponge, the 6/4 nano-sponge was coated on the inner and outer surfaces of a porous PDMS sponge^6^ by dip coating, which showed superhydrophobicity in air and superoleophilicity underwater. Oil (n-hexane) absorption procedure to 6/4 NS/p-PDMS was almost the same as shown in Fig. 1d. Even after full oil absorption (Fig. 4a), many bubble nuclei were still observed inside and on the surface of the NS/p-PDMS. As the UV was irradiated, oil was dragged along with the bubbles in the upward direction because the increased buoyancy, which was due to the continuous generation and coarsening of bubbles, exceeded the lowered interfacial adhesion energy between the oil and the nano-sponge by the UV-activated TiO_2_. Then, the oil was finally detached with the bubbles from the NS/p-PDMS, as shown in Fig. 4c (Supplementary Movie 4). It is worth noting that UV light at the wavelength of 300 nm can penetrate through p-PDMS and NS/p-PDMS (thickness ~5 mm) up to ~50% and ~8% of transmittances, respectively. Intrinsic PDMS transmittance and porous structure enabled all TiO_2_ NPs within NS/p-PDMS to be activated by UV irradiation (Supplementary Fig. S8).

**Figure 3:**
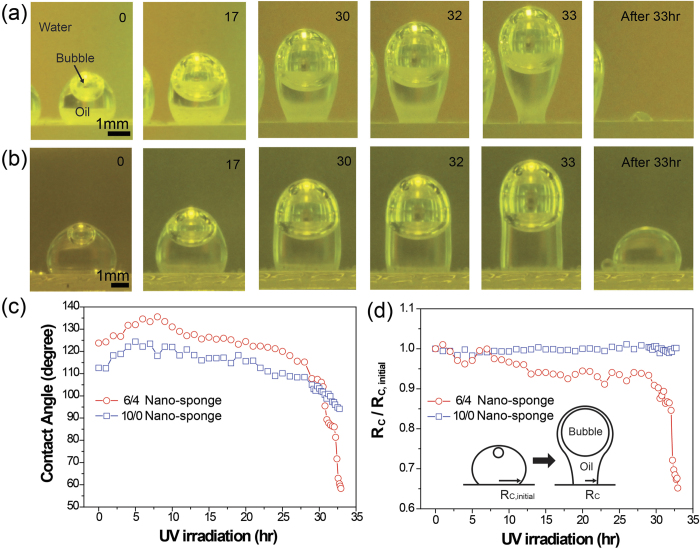
Spontaneous growth of bubbles within an oil droplet and oil/bubble release behavior on the surface of the nano-sponge underwater with UV irradiation: A heavy oil (3 M Fluorinert FC-770, density: 1.793 g/ml) release behaviors from the nano-sponges with hydrocarbon/TiO_2_ volume ratios of (**a**) 6/4, (**b**) 10/0 with UV irradiation for 0, 17, 30, 32, and 33 hr, as well as after 33 hr. (**c**) Underwater oil CA and (**d**) ratio of the oil contact radius (*R*_*C*_) to the original oil contact radius (*R*_*C, initial*_) for the 6/4 and 10/0 nano-sponges with UV irradiation.

**Figure 4:**
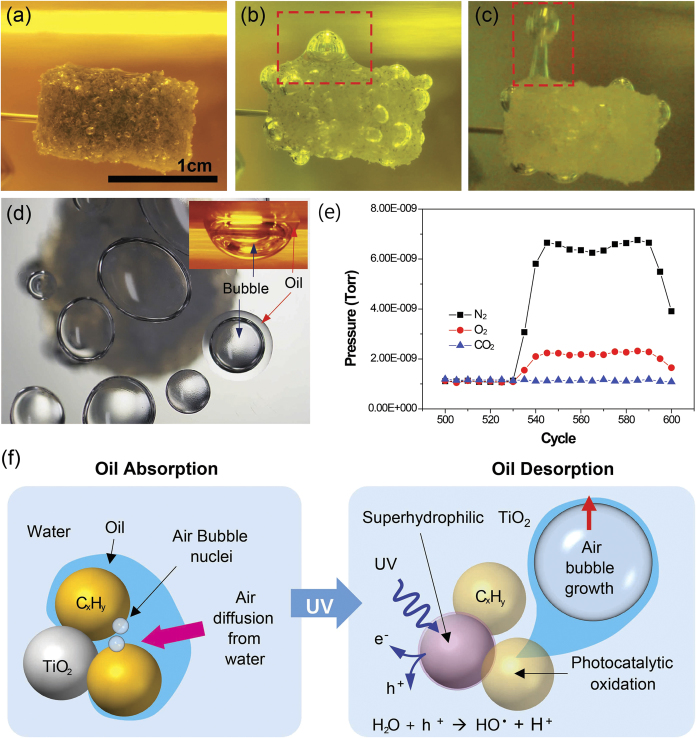
Spontaneous oil (n-hexane, dyed in blue) absorption/desorption of a 6/4 NS/p-PDMS during UV irradiation, and the origin of bubble growth with the oil desorption mechanism. Oil desorption with UV irradiation time (**a**) 0 hr and (**b**) 8 hr, respectively. (**c**) Snapshot of oil/bubble release (~24 hr). (**d**) Top view of the collected oil/bubbles. The inset shows the side view of the collected oil/bubble. (**e**) Mass spectroscopy analysis of collected gas bubbles. (**f**) Mechanism of bubble growth and oil desorption with UV-responsive TiO_2_ Scale bar, 1cm (a-d).

Because continuous bubble growth was observed on the surface of the nano-sponge, as shown in Figs 3 and 4, the bubbles were analyzed for their chemical composition. Rising bubbles that are released with oils were collected by covering the transparent container over the oil-absorbed NS/p-PDMS, as shown in Fig. 4d. As the duration of UV irradiation increased, the number of desorbed oil/bubbles increased; subsequently, the number of bubbles trapped at the bubble/oil collecting surface increased. The collected bubbles were analyzed using mass spectroscopy, confirming that the bubbles were composed of oxygen (17.3%) and nitrogen (82.7%), similar to the contents in air (Fig. 4e). Through the control experiment using deaerated DI water (Supplementary Fig. S9 and S10), it was found that the bubble source originated from dissolved oxygen and nitrogen in the water, indicating that bubble growth was a diffusion-controlled phenomenon. Due to the pre-existing air bubble nuclei in the oil droplets, bubble growth could have occurred by molecular diffusion from the DI water to the air bubble, even at low levels of gas supersaturation in the water^46^. Furthermore, it has been discussed that oil having high gas solubility acts as a gas storage trap; therefore, the mass transfer of oxygen or nitrogen molecules could be enhanced through the oil layer between the air bubble and water phases^47^,^48^. Thus, there is an increased driving force for dissolved air to diffuse through the oil. The bubbles in oil can grow larger due to lower oil surface tensions (organic liquids including oils: 10 ~ 30 mN/m) than the surface tension of water (72.8 mN/m). It is concluded that the mechanism of oil desorption is a combined effect of air bubble growth, wetting transition, and PCO by the UV-responsive TiO_2_, as shown in Fig. 4f.

### Recyclable oil absorption and desorption

**Figure 5:**
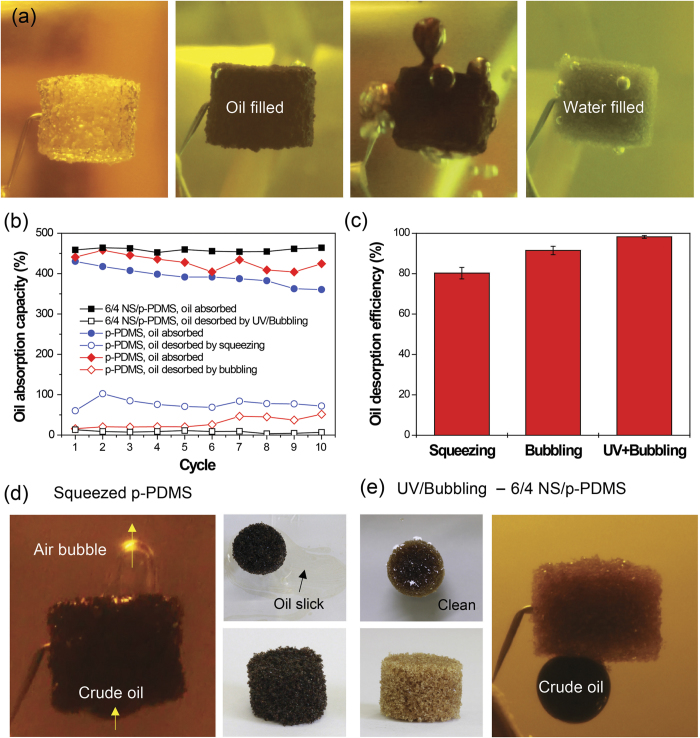
Crude oil absorption and air bubble-driven desorption of a 6/4 NS/p-PDMS sponge during UV irradiation. (**a**) Sequence of underwater crude oil absorption and desorption with UV irradiation and air bubbling. (**b**) Oil absorption/desorption capacities and recyclabilities of the NS/p-PDMS (desorption by UV/bubbling) vs. a p-PDMS (desorption by squeezing or bubbling). (**c**) Oil desorption efficiency: bubbling and UV irradiation on the NS/p-PDMS sponge vs. squeezing the p-PDMS for oil desorption. (**d**) Underwater oleophilic nature of the p-PDMS after squeezing. (**e**) Underwater oleophobic nature of the 6/4 NS/p-PDMS after UV/bubbling. The diameter of each sponge is same as 1.5 cm.

To function as a promising smart material for oil absorption/desorption, the oil absorption capacity and recyclability are the key requirements for practical oil clean-up applications. Thus, the absorption capacity and recyclability of a 6/4 NS/p-PDMS and a p-PDMS were compared using crude oil with different desorption methods of UV/bubbling, only bubbling, and simple squeezing, as shown in Fig. 5. There have been several reports showing that gas bubbles remove contaminants, such as chemicals, powders, and oils, with high efficiency by several gas flotation techniques^49^. Thus, air bubbling was externally supplied to the 6/4 NS/p-PDMS during UV irradiation, resulting in a similar effect to spontaneous bubble formation and growth from dissolved air in water, but with an enhanced oil desorption rate. Figure 5a shows the typical sequence for crude oil absorption and desorption by UV/bubbling: air-filled NS/p-PDMS before oil absorption, oil-filled NS/p-PDMS, desorption of oil, and water-filled NS/p-PDMS after full desorption. During the recycling test, the oil absorption capacity and oil desorption efficiency were determined by weighing the sponges and were calculated as follows:


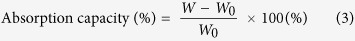



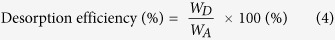


where *W*_*0*_ and *W* are the weight of the sponges before and after oil absorption, respectively. *W*_*A*_ is the weight of absorbed oil, and *W*_*D*_ is the weight of desorbed oil.

After crude oil desorption, the NS/p-PDMS and p-PDMS were dried in a vacuum for several hours to evaporate the infiltrated water and residue of crude oil, respectively. The oil absorption capacity of the NS/p-PDMS was slightly higher due to the increased porosity of the hydrocarbon and TiO_2_ NPs, compared with that of the p-PDMS (459% and 430%, respectively). However, the oil absorption capacity of the NS/p-PDMS was maintained at 459 ± 4% after 10 cycles, whereas that of the p-PDMS gradually reduced from 430% (441%) to 361% (424%) by squeezing (bubbling). Regarding the oil desorption efficiency, with only UV irradiation on the 6/4 NS/p-PDMS, 65% of oil was desorbed for 48 hours due to slow bubble growth. However, with only air bubbling without UV irradiation, 91 ± 2% of absorbed oil was desorbed from the p-PDMS sponge within 1 hour while the oil residues were continuously increased with the number of cycling. The oil desorption by squeezing was 80 ± 3%. According to other researchers, p-PDMS maintained their absorption capacity during recycling tests by squeezing. However, they cleaned the p-PDMS to remove the oil residues using other chemical solvents such as ethanol^6^. This rinsing process itself caused secondary contamination and required effort to dispose of the chemicals. With together of UV/bubbling, the oil residues could be mostly removed and desorption efficiency was as high as 98 ± 1% in the 6/4 NS/p-PDMS sponge as shown in Fig 5c. As discussed earlier, wetting transition under UV irradiation would occurred at the interface between the absorbed oil and the NS/p-PDMS surface, which resulted in almost perfect desorption even after 10 cycle tests. As shown in Fig. 5d, crude oil slicks continuously appeared from the p-PDMS (black color, containing greater than 19% oil) on the water surface even after squeezing and subsequent drying in a vacuum. On the other hand, the NS/p-PDMS sponge showed no slick due to almost complete desorption of the oil during UV/bubbling (yellow color after water evaporation, Fig. 5e). The underwater oil wettability on the NS/p-PDMS was altered to superoleophobicity as the oil was replaced with infiltrated water during UV/bubbling (Fig. 5e and Supplementary Movie 5). For the reuse of the sponge, the NS/p-PDMS sponge restored its original wettability from underwater superoleophobicity to oleophilicity with the sequential process of evaporating the infiltrated water and storage in the dark. Therefore, the oil desorption method of UV triggered wetting transition, when using with bubbling together, had a superior capability for oil clean-up and recyclability without secondary contamination, compared with simple squeezing of the p-PDMS sponge.

## Discussion

A smart approach to control the oil absorption/desorption of the nano-sponge has been demonstrated by the simple mixing of a hydrophobic hydrocarbon and photo-responsive, hydrophilic TiO_2_ NPs. The role of the hydrophobic nature of the hydrocarbon NPs was to selectively absorb only the oil from water. Then, a UV-induced wetting transition by TiO_2_ NPs from a mild oleophilic state to an underwater superoleophobic state released the absorbed oil from the nano-sponge with UV irradiation. This was further demonstrated by the NS/p-PDMS with high oil absorption/desorption efficiency without the occurrence of secondary pollution. Because materials for smart control of oil absorption/desorption are not limited only to hydrocarbons and TiO_2_ NPs, any type of mixture of hydrophobic and hydrophilic materials with photo-switchable wettability could be used to realize oil-water separation and oil spill clean-up. Furthermore, nano-sponge is expected to separate the various type of emulsions, due to the wide range of tunable wettability with several tens of nanometer scale pores for practical application for emulsion separation.

## Methods

### Fabrication of the nano-sponge

Hydrocarbon^11^ and TiO_2_^50^ NPs were synthesized by glow discharge and chemical vapor synthesis, respectively. Because both hydrocarbon and TiO_2_ NPs have similar diameters (~30 nm in average), hydrocarbon/TiO_2_ NPs were mixed at the volume ratios of 10/0, 8/2, 6/4, 4/6, 2/8, and 0/10 (Supplementary Fig. S1 and Table S1). The mixed NPs were dispersed in isopropyl alcohol (IPA) and were ultrasonicated. IPA solutions containing hydrocarbon/TiO_2_ NPs with different volume ratios were coated by drop casting on glass slides. The slides were left to evaporate and were used to measure both the water and oil contact angle in the air and the oil contact angle underwater.

### Fabrication of the NS/p-PDMS

A porous PDMS was selected as the membrane for oil absorption-desorption experiments under UV irradiation. Because PDMS has high transmittance and low absorption under UV irradiation^51^, TiO_2_ NPs within a nano-sponge coated porous PDMS can act as a photocatalyst for hydrocarbon decomposition and display a wettability transition for oil absorption and desorption. The porous PDMS was fabricated using the sugar templating method^6^. The fabricated porous PDMS was dipped into IPA solutions containing hydrocarbon/TiO_2_ NPs, ultrasonicated for 10 min, and finally dried in a vacuum chamber.

### Characterization methods

A scanning electron microscope (SEM, Inspect F50, FEI) operated at an accelerating voltage of 10 keV, which was used to observe the morphology and configuration of hydrocarbon/TiO_2_ NPs. All of the specimens for SEM observation were coated with Pt to reduce the charging effect. Compositional analysis was performed using energy-dispersive X-ray spectroscopy (EDS) to confirm the compositional ratio of the mixed NPs. High-resolution transmission electron microscopy (HR-TEM) and electron energy loss spectroscopy (EELS) were used to confirm the distribution of hydrocarbon and TiO_2_ NPs, with the corresponding compositional mapping of carbon, oxygen, and titanium. CA was measured by sessile drop method, after 5 seconds from the liquid drop placement on the surface for stabilization, and it was kept constant for each CA measurement. The UV-responsive wettability of the nano-sponge coated glass slides was characterized by measuring the CA of deionized (DI) water and oil (n-hexane) droplets in the air and underwater, respectively. After each nano-sponge was UV-irradiated for 2 hours (18 W Hg lamp with wavelength 280 ~ 360 nm and irradiance of 51 μW/cm^2^) with a distance of ~10 cm between the UV light source and the specimen, superhydrophilicity in the air and superoleophobicity underwater were realized. For the CA measurements of DI water in air, droplets of approximately 5 μL were gently deposited on the substrates, using a microsyringe. To measure the underwater oil CA, the nano-sponge coated glass slides were inverted and placed in a transparent container filled with DI water. Then, oil drops were gently inserted by a syringe needle to the coated surface. All of the measurements were recorded using a digital camera at room temperature. Two different types of oils, n-hexane (density of 0.6548 g/ml) as a light oil and 3 M Fluorinert FC-770 lubricant (density of 1.793 g/ml) as a heavy oil, were tested for the release of the adhered oil under UV irradiation. The effects of TiO_2_ on the wetting transition and the oil release behaviors with UV irradiation were evaluated by comparing the oil desorption from the 10/0 and 6/4 (hydrocarbon/TiO_2_) nano-sponges. X-ray photoelectron spectroscopy (XPS, PHI 5000 VersaProbe (Ulvac-PHI)) was used to determine the binding energy of the 6/4 nano-sponge before and after UV irradiation. The background pressure was decreased to ~6.7 × 10^−8^ Pa. The XPS data were collected using monochromatized Al Ka radiation at 1486.6 eV. All of the binding energies were calibrated using the C 1s peak at 284.6 eV. Gas bubbles grown on the surface of the nano-sponge during UV irradiation were collected under water. To prevent interference by other gas molecules in the atmosphere, the collected gas bubble were directly injected to a Quadrupole mass spectroscopy (HPR20, Hiden Analytical) with He as a carrier gas; the sample were analyzed to identify and quantify the gas phases. UV-visible spectrophotometer (Varian Cary 100) was used to investigate the transmission of UV light into the NS/p-PDMS (data available in Supplementary Information). Additional air bubble flow was supplied to the NS/p-PDMS to enhance the oil desorption rate, after crude oil (32.8° API, density: 860 kg/m^3^) absorption under UV irradiation.

## Additional Information

**How to cite this article**: Kim, D. H. *et al.* UV-responsive nano-sponge for oil absorption and desorption. *Sci. Rep.* **5**, 12908; doi: 10.1038/srep12908 (2015).

## Supplementary Material

Supplementary Information

Supplementary Movie 1

Supplementary Movie 2

Supplementary Movie 3

Supplementary Movie 4

Supplementary Movie 5
