# Supplementary material for: UV-responsive nano-sponge for oil absorption and desorption
Source: Sci Rep. 2015 Aug 11;5:12908. doi: 10.1038/srep12908 (PMC4531332; doi:10.1038/srep12908)
Supplement: Supplementary Information [file srep12908-s1.pdf]

Supplementary Information:

UV-responsive nano-sponge for oil absorption and desorption

Do Hyun Kim[1,2], Min Chan Jung[3], So-Hye Cho[2], Sang Hoon Kim[2], Ho-Young Kim[3], Heon Ju Lee[2], Kyu Hwan Oh[1], and Myoung-Woon Moon[2,*]

1. Department of Materials Science and Engineering, Seoul National University, Seoul 151-742, Republic of Korea

2. Institute of Multidisciplinary Convergence of Matter, Korea Institute of Science and Technology, Seoul 136-791, Republic of Korea

3. Department of Mechanical and Aerospace Engineering, Seoul National University, Seoul 151-744, Republic of Korea

*Corresponding Author: mwmoon@kist.re.kr

Nano-sponge preparation

Hydrocarbon NPs and TiO_2 nanoparticles (NPs) were synthesized by the methods in experimental section. Both of hydrocarbon and TiO2 NPs were packed within cylinder tubes with a diameter of 11 mm up to height of 1.5 mm. For the same volume, each tube of packed NPs was weighed. The hydrocarbon NPs weighed 0.0518 g, and the TiO_2 NPs weighed 0.080 g due to their different atomic densities (1.2~2.2 g/cm^3 for the hydrocarbon[1], 3.89 g/cm^3 for anatase TiO_2[2]). Each nano-sponge was prepared by simply mixing in isopropyl alcohol (IPA) solution at the given volume mixing ratio (Table S1). The surface microstructure and atomic composition of each nano-sponge was characterized by scanning electron microscopy (SEM) and energy-dispersive X-ray microscopy (EDS) as shown in Figure S1 and Table S1.

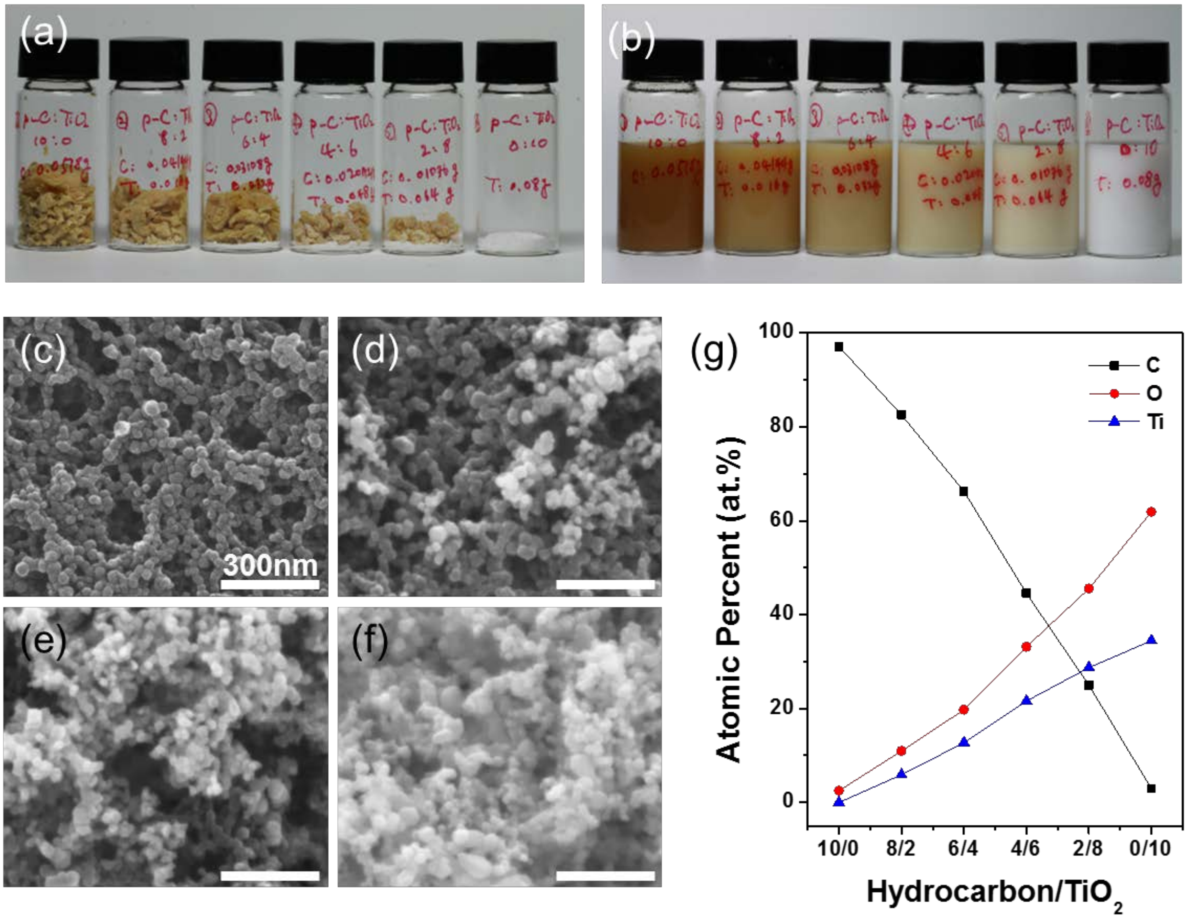

Figure. S1. Mixture of a hydrophobic hydrocarbon and hydrophilic TiO_2 nanoparticles and the surface morphology and chemical composition of the nano-sponge using scanning electron microscopy (SEM) and energy-dispersive X-ray spectroscopy (EDS). Photograph of hydrocarbon/TiO_2 NPs (a) before and (b) after mixing in an IPA solution. SEM images of nano-sponges (hydrocarbon/TiO_2 NPs) with volume

ratios of (c) 10/0, (d) 6/4, (e) 4/6 and (f) 0/10. (g) Atomic composition of nano-sponges with different mixing volume ratios measured with EDS.

Table S1. Mixing ratio of nano-sponges and the atomic composition of the nanoparticles determined using energy-dispersive X-ray spectroscopy.

**Hydrocarbon**	**TiO_2**	**Mixing Ratio**	**Atomic composition (at. %)**		
Weight (g)	Weight (g)	Volume ratio	C	O	Ti
0.0518	0	10/0	97.11	2.5	0
0.04144	0.016	8/2	82.58	10.98	5.93
0.03108	0.032	6/4	66.28	19.74	12.74
0.02072	0.048	4/6	44.56	33.15	21.61
0.01036	0.064	2/8	25	45.57	28.71
0	0.08	0/10	2.94	61.92	34.47

Size distribution of hydrocarbon and TiO_2 nanoparticles and pores

Average diameters of hydrocarbon and TiO_2 NPs are 31±9 nm and 30±9 nm, respectively. Pore diameters were obtained by measuring the pore area through SEM image analysis (threshold method in image J) assuming pore shape as circle. Average pore size of hydrocarbon and TiO_2 NP are 67±52nm and 60±55nm, respectively. The pore fraction of each sample showed similar values of 41.3% in average regardless of mixing ratio of hydrocarbon and TiO_2 NP.

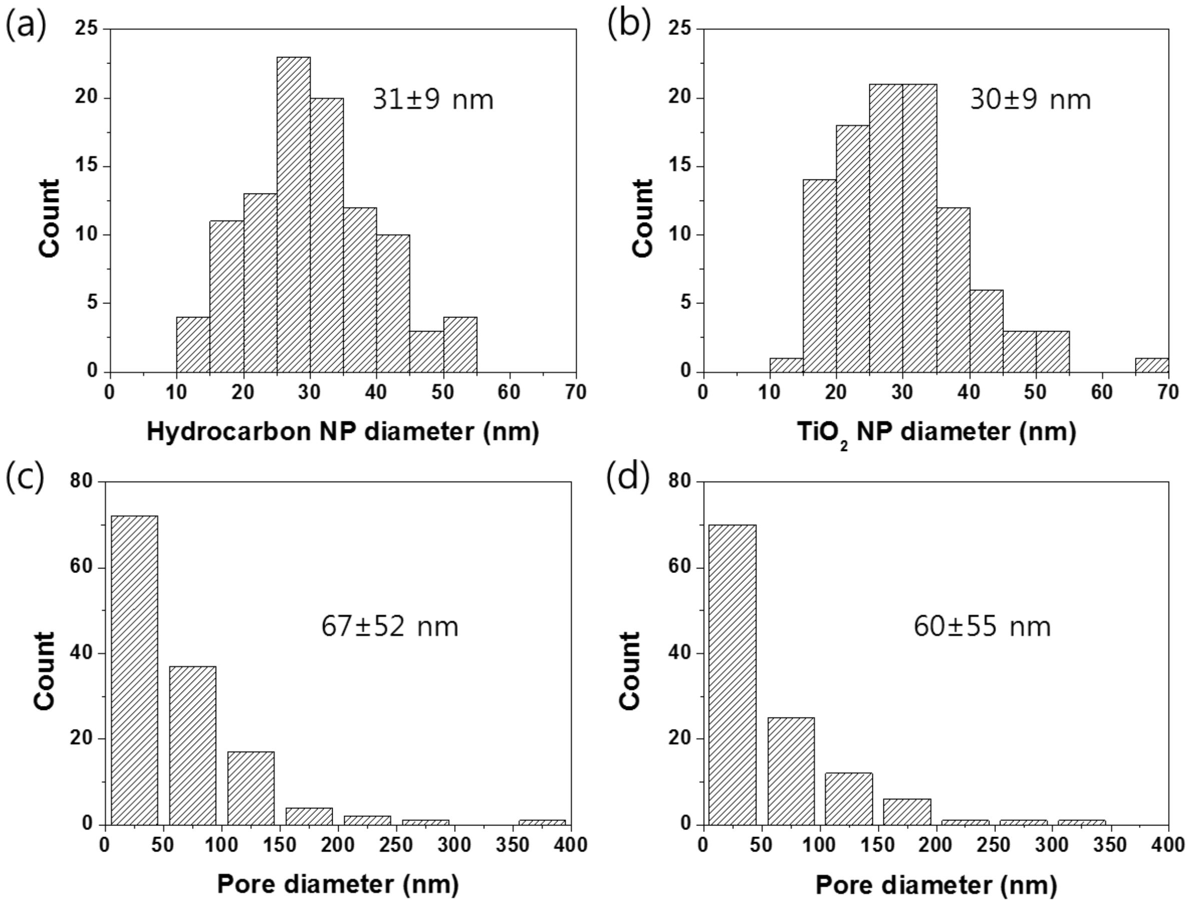

Figure S2. Size distributions of (a) hydrocarbon and (b) TiO_2 nanoparticles and pore size distributions in (c) 10/0 (hydrocarbon/TiO_2) and (d) 0/10 nano-sponge.

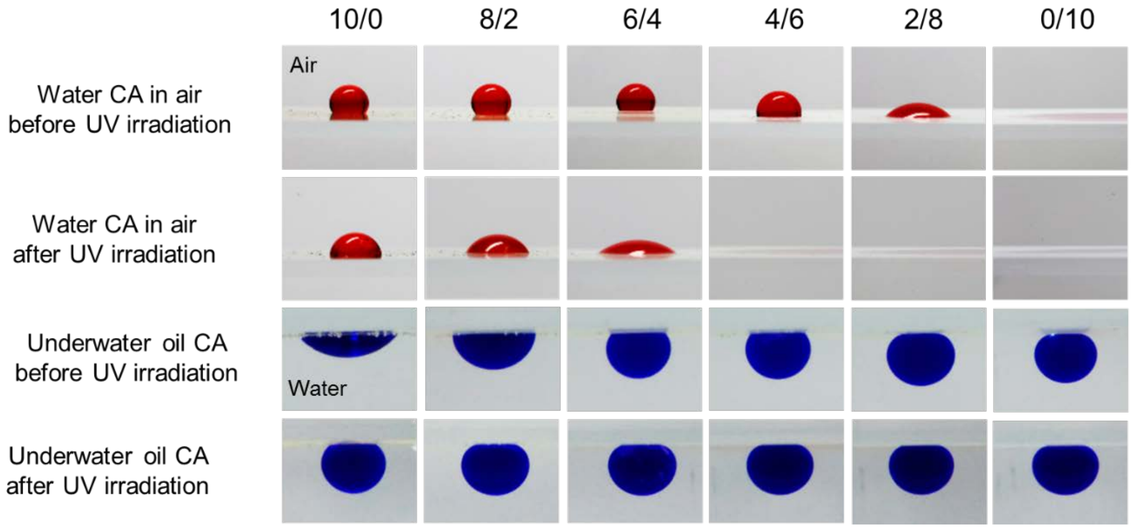

Figure. S3. Water CA in air and oil (n-hexane) CA underwater on nano-sponges for each volume ratio (hydrocarbon/TiO_2: 10/0, 8/2, 6/4, 4/6, 2/8, 0/10) before/after UV irradiation for 2 hr.

Contact angle for calculation of work of adhesion

Theoretically, underwater oil CA in Young-Dupré equation means equilibrium (Young) CA. Apparent, advancing, and receding CAs of 6/4 NS were measured as a representative sample before/after UV irradiation, and the measured data are shown as follows.

Table S2. Apparent, advancing, and receding CAs of 6/4 nano-sponge before and after UV irradiation.

6/4 nano-sponge	Apparent CA	Advancing CA	Receding CA
Before UV	114 ± 7 °	122 ± 1 °	~ 0 °
After UV	158 ± 2 °	162 ± 1 °	153 ± 1 °

Even though advancing CA should be used to calculate the minimum work of adhesion, we assumed that the apparent CA is equivalent to advancing CA for the following reasons;

First, the measured advancing CAs of 6/4 NS are very close to the apparent CA, with deviation of less than 8°. According to the CAs in the Table S3, it showed high CA hysteresis before UV irradiation. However, CA hysteresis became less than 10°, and the advancing CA was very similar to the apparent

CA after UV irradiation. Second, hydrocarbon & TiO_2 nanoparticles having similar diameter (~30 nm) resulted in the almost constant pore size (~63 nm) and fraction (~41.3%) in average, regardless of the mixing ratio of hydrocarbon and TiO_2 NPs. Based on these results, work of adhesion that was calculated by using apparent CA would show similar trend with minimum work of adhesion calculated by using advancing CA.

Effect of salt (NaCl) on contact angle measurement

The stability of nano-sponge in sea water was evaluated by measuring the underwater oil contact angle of 6/4 nano-sponge in salt water (3.5% dissolved NaCl in DI water), before and after UV irradiation. The apparent, advancing, and receding CAs in DI water and salt water showed very similar values, and the effect of salt on underwater CA was negligible.

Table S3. Contact angles (apparent, advancing, and receding CAs) of 6/4 nano-sponge: effect of salt before and after UV irradiation

6/4 nano- sponge		Apparent CA	Advancing CA	Receding CA
DI water	Before UV	114±7°	123±2°	~ 0°
	After UV	158±2°	161±1°	153±1°
Salt water	Before UV	111±4°	125±3°	~ 0°
	After UV	160±2°	162±4°	160±2°

Calculation of interfacial tension between oil and water

As addressed in the manuscript, measurements of the interfacial tension of n-hexane and FC-770 were needed to obtain the spreading coefficients, which explained bubble encapsulation within oil droplets. The pendant drop method was used to measure the interfacial tension between the oil (n-hexane, FC-770) and water (density: 0.999 g/ml). The equatorial diameter (D) and the diameter (d) at the distance D from the top of the drop were experimentally measured, as shown in Fig. S4. The interfacial tension was calculated using the following equation[3].

$$\gamma = \frac{\Delta\rho g D^2}{H} \quad (1)$$

The shape-dependent parameter (H) depends on the shape factor S=d/D. The values of 1/H were calculated using the following empirical formula[3].

$$\frac{1}{\mathrm{H}} = \frac{\mathrm{B}_4}{\mathrm{S}^{\mathrm{a}}} + \mathrm{B}_3\mathrm{S}^3 - \mathrm{B}_2\mathrm{S}^2 + \mathrm{B}_1\mathrm{S} - \mathrm{B}_0 \quad (2)$$

Because the values of S for FC-770 (0.577) and n-hexane (0.475) were in the range of 0.46~0.59, the empirical constants a, B_4, B_3, B_2, B_1, and B_0 were used as 2.59725, 0.31968, 0, 0.46898, 0.50059, and 0.13261, respectively[3]. The measured data and calculated interfacial tension in water were listed as follows.

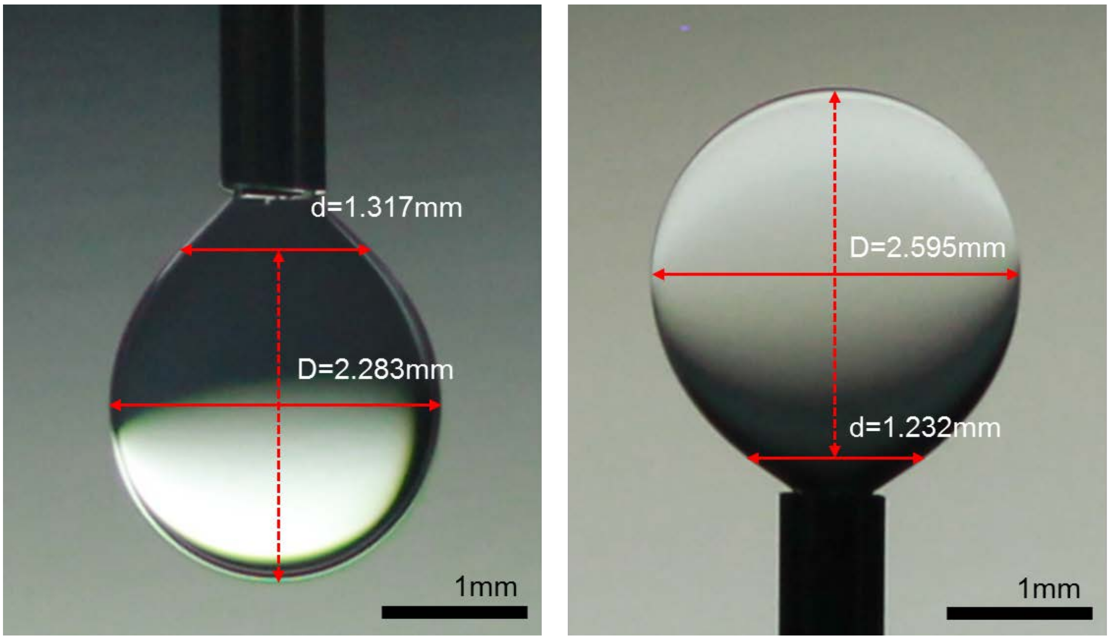

Figure S4. Calculation of the interfacial tension of Fluorinert FC-770 (left, density: 1.793 g/ml) and n-hexane (right, density: 0.6548 g/ml), which were measured under water.

Table S4. Measured parameters for the calculation of the interfacial tension between oil and water.

Oil	*D* (mm)	*d* (mm)	*S=d/D*	γ (mN/m)
FC-770	2.283	1.317	0.577	54.2
n-hexane	2.595	1.232	0.475	50.2

Calculation of spreading coefficient of oil

These interfacial tension values were used to calculate the spreading coefficient[4] of oil as follows.

$$S_o = \gamma_{wg} - \gamma_{ow} - \gamma_{og} \tag{3}$$

where γ_{wg} is the water-gas surface tension, γ_{ow} is the oil-water interfacial tension and γ_{og} is the oil-gas surface tension. The spreading coefficient (S_o) is a measure of one fluid spontaneously spreading relative to another in the third phase. Therefore, as the value of S increases, the tendency to spread increases. The surface tension values of water (γ_{water}: 72.8 mN/m), n-hexane (γ_{hexane}: 18.4 mN/m), FC-770 ($\gamma_{FC\text{-}770}$: 14.8 mN/m) and the calculated interfacial tension values with water for n-hexane ($\gamma_{hexane\text{-}water}$: 50.2 mN/m) and FC-770 ($\gamma_{FC\text{-}770\text{-}water}$: 54.2mN/m) were used for calculating the spreading coefficient. Through eq. (3), S_o values for n-hexane and FC-770 were calculated as +4.2 and +3.8 mN/m, respectively.

Spontaneous growth of bubbles within an oil droplet under UV irradiation

The underwater oil CAs were monitored *in-situ* under UV irradiation on the nano-sponge coated glass slide, as shown in Fig. S5. A light oil droplet (n-hexane, density: 0.6548 g/ml) was placed on the 6/4 nano-sponge coated glass slide, in which the mixing ratio was selected to show high adhesion contrast; underwater oleophobicity with high adhesion before UV irradiation and superoleophobicity with low adhesion after UV irradiation. As the nano-sponge coated glass was immersed into water, air bubbles with an average volume of 0.015 mm^3 were trapped on the surface of the nano-sponge, which were mainly captured by the hydrophobic hydrocarbon NPs. As the oil droplet contacted the surface, the nano-sponge was partially wetted by the oil, resulting in a CA of 113°. Simultaneously, air bubbles trapped on the surface of nano-sponge were encapsulated within oil droplets due to the positive oil spreading coefficient, S_o (+4.2 mN/m for n-hexane, Supplementary Fig. S4 and Table S2). Interestingly, air bubbles encapsulated within oil droplets continuously grew with the increasing UV irradiation time, and the oil CA increased from 113° to 157°, which indicated that a wetting transition occurred from mild oleophobicity to superoleophobicity underwater. In the beginning of the UV irradiation, several small bubbles began to grow and coarsen. Then, these bubbles became one larger bubble and provided increased buoyancy to the bubble containing the oil droplet until it finally released the oil droplet.

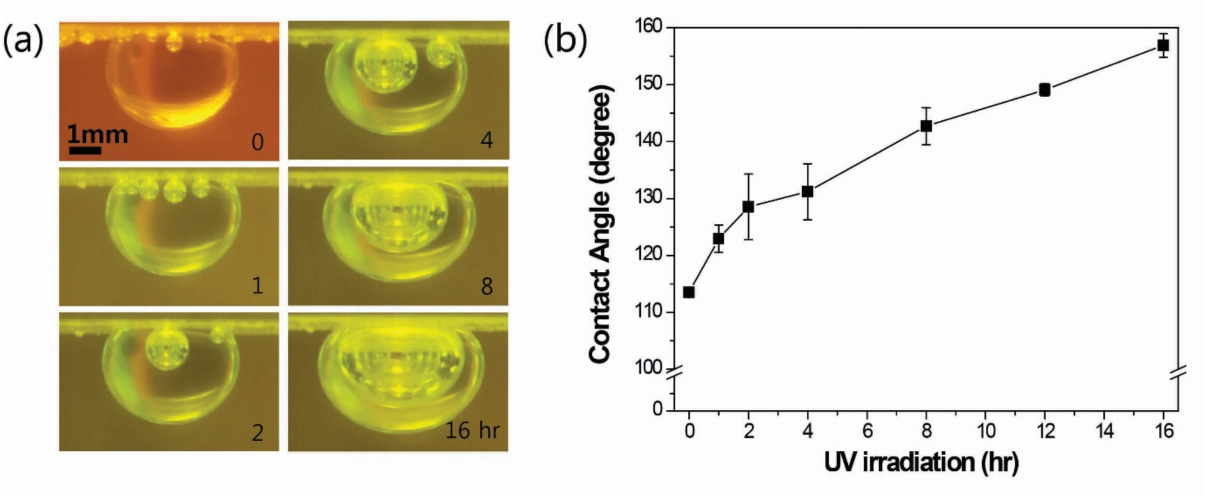

Figure S5. Spontaneous growth of bubbles within an oil droplet on the surface of nano-sponge underwater with UV irradiation. (a) Bubble growth within a light oil (n-hexane, density: 0.6548 g/ml) droplet in contact with 6/4 nano-sponge underwater with increasing UV irradiation times of 0, 1, 2, 4, 8, and 16 hr and (b) underwater oil CA measured from (a).

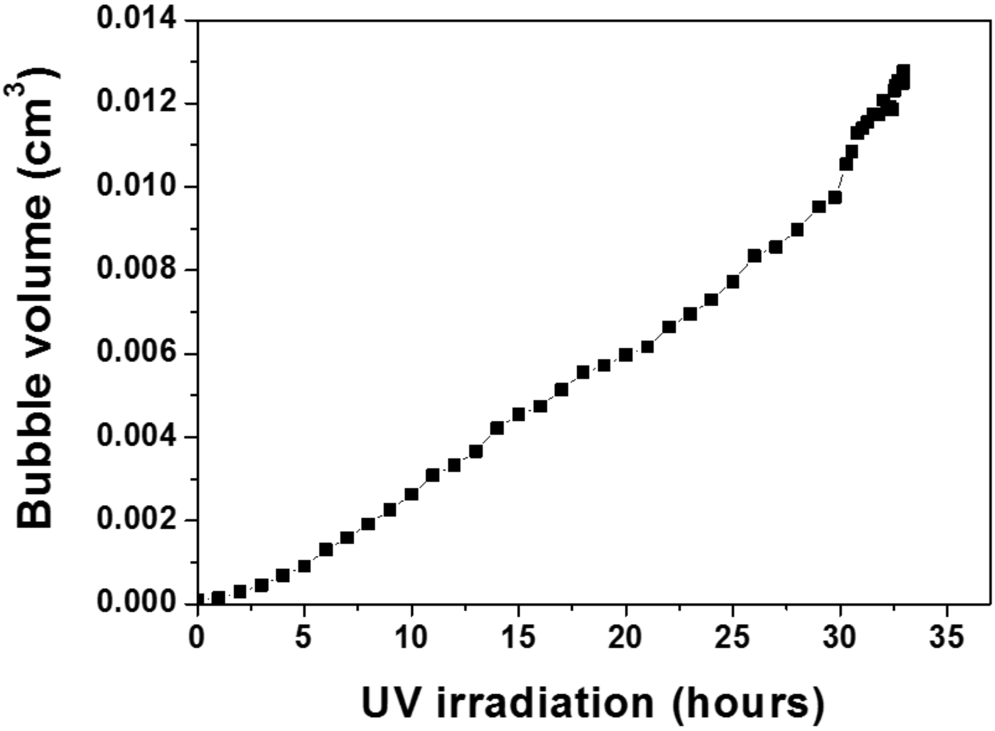

Figure S6. Changes in bubble volume within oil (FC-770) droplet on the 6/4 nano-sponge during UV irradiation.

Vertical force balance at the oil-solid interface during UV irradiation Regarding the forces acting at the oil-solid interface, the vertical forces of buoyancy, pressure, and surface tension, can be expressed as follows

$$F_B = F_{B,bubble} + F_{B,oil} = \rho_w g V_b + \rho_w g V_o - \rho_o g V_o \quad (4)$$

$$F_P = \Delta P(\pi R_c^2) = \left(\frac{2\gamma_{ow}}{R} - (\rho_w - \rho_b) g h_1 - (\rho_w - \rho_o) g h_2\right) \times (\pi R_c^2) \quad (5)$$

$$F_S = \gamma_{ow}(2\pi R_c)\sin\theta \quad (6)$$

$$F_S = F_P + F_B \quad (7)$$

where F_B, $F_{B,bubble}$, $F_{B,oil}$, F_P, F_S, γ_{ow}, R_C, θ, ρ_w, ρ_o, g, V_b, V_o, R, h_1, and h_2 are the force induced by buoyancy, buoyancy forces of the bubble and oil, pressure force due to excess pressure (ΔP) inside the oil and bubble acting on the contact area, surface tension force, oil-water interfacial tension, oil contact radius, underwater oil CA on solid, the densities of water and oil, acceleration due to gravity, bubble volume, oil volume, radius of curvature at the top of the oil droplet, bubble height, and height between

the solid surface and bubble bottom, respectively. The magnitude of the excess pressure across the curved oil-water interface from the Young-Laplace equation is $2\gamma_{ow}/R$. If hydrostatic pressure under gravity is applied, the total excess pressure can be taken as the right hand side of eq. (5).

Because the bubble growth rate was very small, only static forces were considered. Therefore, each force was balanced in eq. (7), and the surface tension force F_S and buoyancy force $F_{B,oil}$ of the oil droplet were responsible for holding the oil on the nano-sponge surface. The pressure, F_P, and bubble buoyancy, $F_{B,bubble}$, forces were responsible for pulling the bubble and oil off from the surface. Each force was non-dimensionalized by dividing the buoyancy force of the oil to illustrate the force exerted for the unit density and unit volume of oil. In the beginning of UV irradiation, F_B was negative (downward direction) due to large oil density and $F_{B,bubble}$ was negligible. As buoyancy force of the bubble increased with the growth of bubble while that of the oil was unchanged, the total buoyancy force acting upward increased (became positive) with UV irradiation. Additionally, the surface tension force (F_S) of the 6/4 nano-sponge working against the buoyancy and the Laplace pressure forces decreased due to receding-induced R_C reduction ($R_B/R_C \approx 2.36$), whereas that of the 10/0 nano-sponge was maintained due to contact line pinning ($R_B/R_C \approx 1.04$), as shown in Fig. S7b, c. At the moment of detachment, F_P would be nearly zero due to the negligible neck radius. Consequently, the remaining oil volumes were determined by the magnitude of the surface tension force acting at the oil-solid interface before oil or bubble detachment.

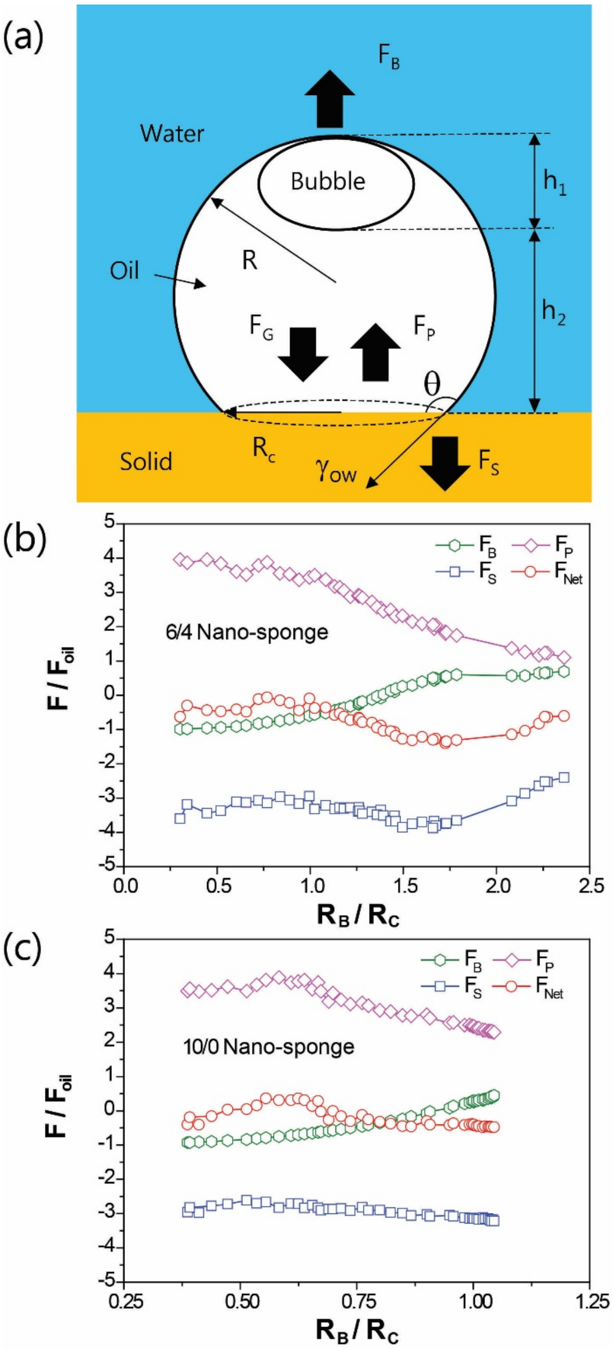

Figure S7. Vertical force components of the surface tension (F_S), pressure force (F_P), and buoyancy (F_B) of the oil/bubble at the oil-solid interface underwater during UV irradiation: (a) schematic illustration of each force component. The buoyancy, pressure force, surface tension force, and net force (F_{NET}) were non-dimensionalized and plotted as bubble radius (R_B)/contact radius (R_C) for (b) 6/4 and (c) 10/0 nano-sponges.

Transmittance of nano-sponge coated porous PDMS

The transmittance of incident UV light through 6/4 NS/p-PDMS was measured by UV-visible spectrophotometer. 6/4 nano-sponge were coated on p-PDMS with thickness of 5 mm and transmittance was compared with that of p-PDMS. It was measured that almost 67% of light can penetrate through p-PDMS in visible regime, and transmittance was even ~50 % at the wavelength of 300 nm. For the sample thickness with 5 mm, transmittance of 6/4 NS/p-PDMS was reduced to ~8 % at the wavelength of 300 nm. The difference in the transmittance represent the absorption by TiO_2 at lower wavelength of 200~300 nm. The result shown in Figure S8 indicates that UV light can penetrate into the inner part of the sponge and activate the photocatalytic oxidation and wettability transition. The light loss at lower wavelength of 200~300 nm represents the absorption by TiO_2.

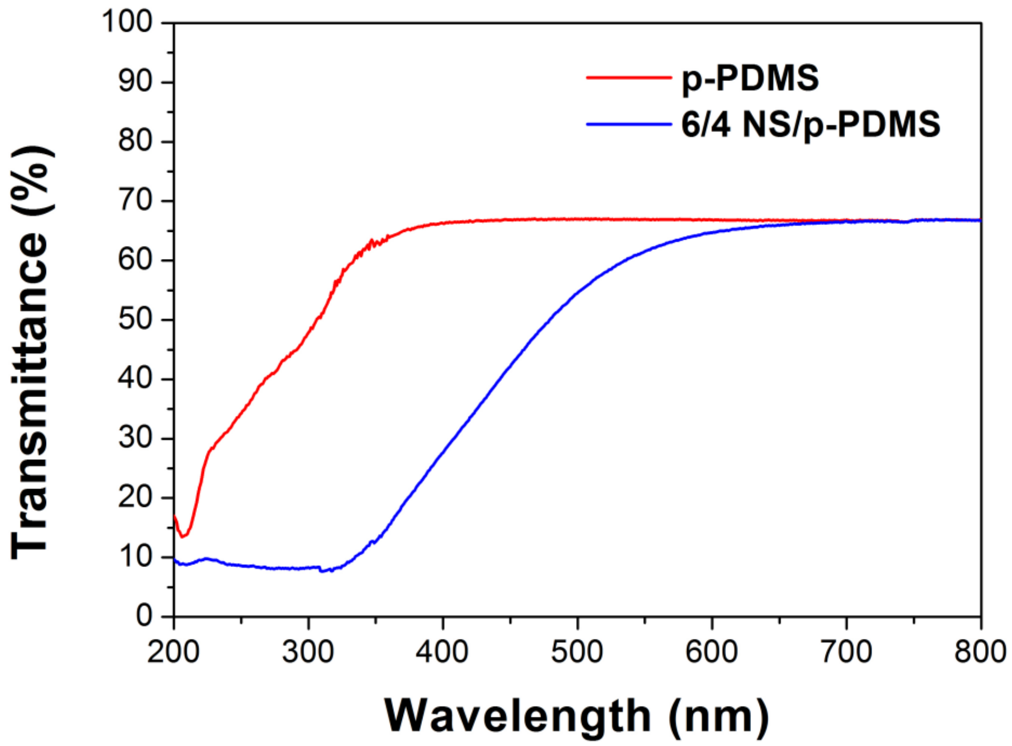

Figure S8. UV-vis spectra of p-PDMS and 6/4 NS/p-PDMS with thickness of 5mm.

Origin of bubble source: Control experiment for bubble formation in oil.

Because gases usually become less soluble in water with increasing temperature, DI water was heated to 100℃ for deaeration, sealed, and cooled to room temperature. Samples of a transparent substrate (glass slide or PET) and nano-sponge coated substrates with various mixing ratios were stored in the

dark and in UV irradiation for several hours, and no bubble was formed in the boiled DI water. When the substrate without nano-sponge was immersed in DI water, formation and growth of bubbles were observed. These control experiments revealed that the source of bubble growth was diffusion from dissolved oxygen and nitrogen in water.

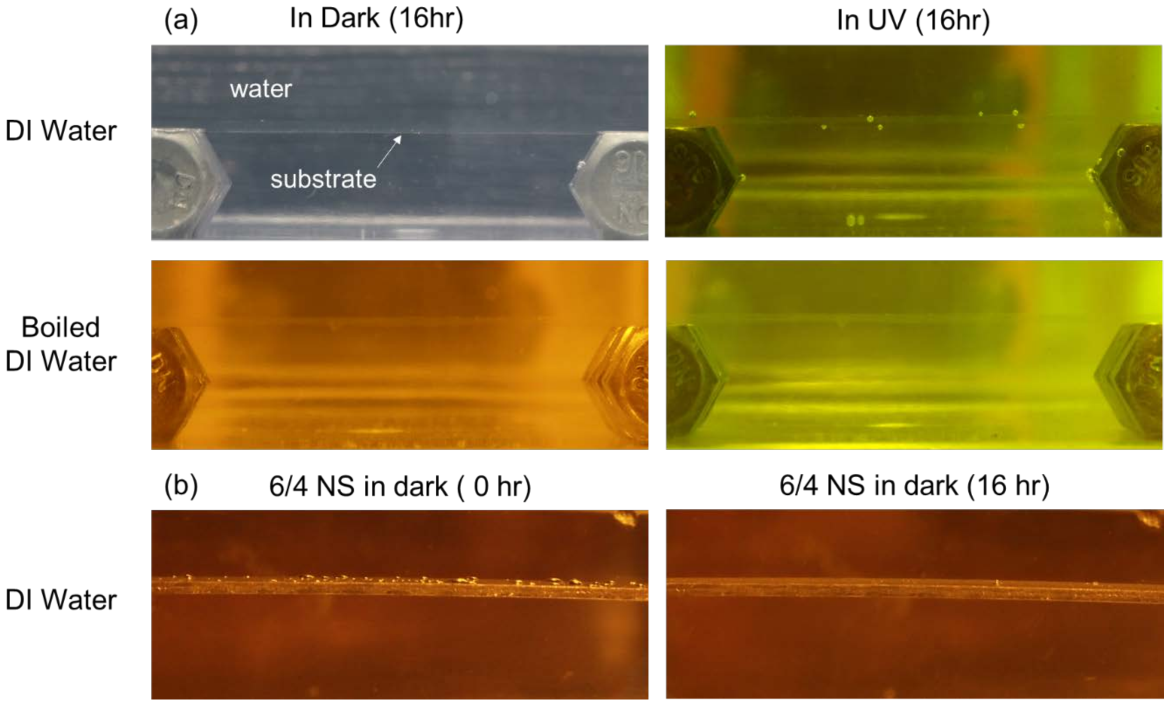

Figure S9. Effect of dissolved air in water on bubble formation and growth. Control experiment: (a) only substrates (glass slide or PET plate) were immersed into water and stored in dark and in UV irradiation for 16 hr with different amounts of dissolved gas in water (DI water/boiled DI water) (b) Storage of 6/4 nano-sponge/PET in DI water for 16 hr in the dark.

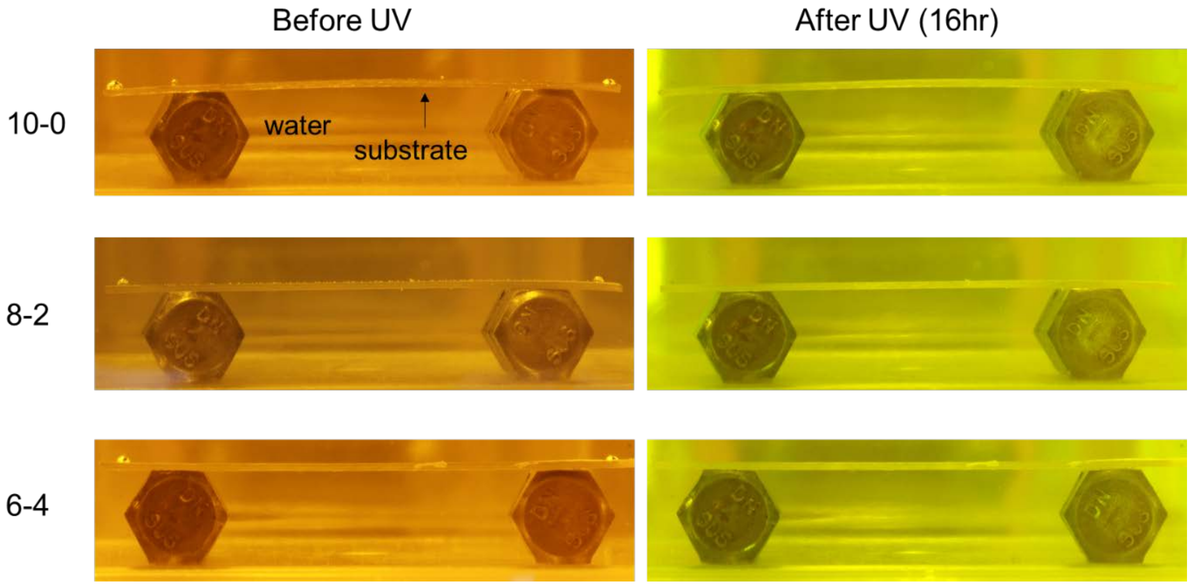

Figure S10. Effect of nano-sponges on bubble formation and growth. Control experiment: each nano-sponge having a different volume ratio (hydrocarbon/TiO_2: 10/0, 8/2, 6/4) coated on a PET plate was immersed in boiled DI water under UV irradiation for 16 hr.

Supplementary Movie 1.

Absorption of crude oil onto the 6/4 nano-sponge/porous PDMS (NS/p-PDMS). As the oil contacted the NS/p-PDMS, it instantaneously absorbed the oil and released the air bubbles trapped inside the porous NS/p-PDMS.

Supplementary Movie 2.

Desorption of crude oil from the 6/4 NS/p-PDMS via UV irradiation and supplied air bubble flow.

Supplementary Movie 3.

Detachment of an oil (FC-770) droplet with an air bubble from the 6/4 nano-sponge surface, leaving only a small amount of oil residue after UV irradiation.

Supplementary Movie 4.

Desorption of oil (n-hexane) from 6/4 NS/p-PDMS with spontaneous bubble growth under UV irradiation.

Supplementary Movie 5.

Wettability transition of 6/4 NS/p-PDMS after UV irradiation, which replaces the oil with water. The sponge was fully filled with water, showing that most of the absorbed oil was desorbed via UV/bubbling.

References

1. Robertson, J. Diamond-like amorphous carbon. *Mater. Sci. Eng. R.* **37,** 129-281 (2002).

2. Anthony, J. W., Bideaux, R. A., Bladh, K. W. & Nichols, M. C. *Handbook of Mineralogy* Vol. 3 Halides, Hydroxides, Oxides (Mineral Data Publishing, Tucson, Arizona, 1997).

3. Drelich, J., Fang, C. & White, C. L. Measurement of interfacial tension in fluid-fluid systems in *Encyclopedia of Surface and Colloid Science*, 2nd edition, Vol. 3 (Marcel Dekker, New York, 3152-3166, 2002).

4. De Gennes P. G. Wetting: statics and dynamics. *Rev. Mod. Phys.* **57,** 827-863 (1985).
